# Stromal androgen signaling governs essential niches in supporting prostate development and tumorigenesis

**DOI:** 10.1038/s41388-024-03175-1

**Published:** 2024-10-05

**Authors:** June‑Wha Rhee, Yao Mawulikplimi Adzavon, Zijie Sun

**Affiliations:** 1 Department of Medicine, City of Hope Comprehensive Cancer Center, Duarte, CA USA; 2 Department of Cell Biology, Department of Oncology, Montefiore Cancer Center, Albert Einstein College of Medicine, Bronx, NY USA

**Keywords:** Prostate cancer, Cancer microenvironment, Stem-cell niche

## Abstract

Androgens and androgen receptor (AR) mediated signaling pathways are essential for prostate development, morphogenesis, growth, and regeneration. Early tissue recombination experiments showed that AR-deficient urogenital sinus mesenchyme combined with intact urogenital sinus epithelium failed to develop into a prostate, demonstrating a stem cell niche for mesenchymal AR in prostatic development. Androgen signaling remains critical for prostate maturation and growth during postnatal stages. Importantly, most primary prostate cancer (PCa) cells express the AR, and aberrant activation of AR directly promotes PCa development, growth, and progression. Therefore, androgen deprivation therapy (ADT) targeting the AR in PCa cells is the main treatment for advanced PCa. However, it eventually fails, leading to the development of castration-resistant PCa, an incurable disease. Given these clinical challenges, the oncogenic AR action needs to be reevaluated for developing new and effective therapies. Recently, an essential niche role of stromal AR was identified in regulating prostate development and tumorigenesis. Here, we summarize the latest discoveries of stromal AR niches and their interactions with prostatic epithelia. In combination with emerging clinical and experimental evidence, we specifically discuss several important and long-term unanswered questions regarding tumor niche roles of stromal AR and highlight future therapeutic strategies by co-targeting epithelial and stromal AR for treating advanced PCa.

## Introduction

Androgen signaling pathways mediated through the androgen receptor (AR) and androgens play an essential role in prostate formation, growth, and regeneration [1]. During embryogenesis, the AR is first expressed in the urogenital sinus mesenchyme (UGM), and its expression subsequently extends to the urogenital sinus epithelium (UGE) [1, 2]. Early tissue recombination assays showed that AR-deficient UGM combined with intact UGE failed to develop into a prostate [3–5], providing the first line of experimental evidence to demonstrate a stem cell niche role of mesenchymal AR signaling in supporting prostatic early development. Androgen signaling still remains essential during postnatal stages, controlling prostate maturation, growth, and regeneration through paracrine interactions between mesenchymal and epithelial cells [6].

Aberrant activation of androgen and AR-mediated signaling pathways also directly contributes to the pathogenesis of prostate cancer (PCa) [7, 8]. PCa is one of the most common malignancies and the second leading cause of cancer-related death among men, accounting for 30,000 deaths annually in the United States alone [9]. The AR is expressed in most primary PCa cells [7, 8, 10], and its activation by binding of its ligands, androgens, directly promotes PCa development, growth, and progression [7, 8, 11, 12]. Therefore, over the past decades, significant effort has been devoted to determining the intrinsic mechanism underlying AR action in PCa cells [13–15]. However, androgen deprivation therapy (ADT) [16], designed to directly target AR-expressing prostate epithelial tumor cells through either antagonizing the AR or inhibiting androgen production, ultimately fails in almost all of patients, leading to the development of castration-resistant PCa (CRPC), an incurable disease [7, 11].

Given these critical challenges in clinics, it is imperative to reassess the oncogenic roles of AR signaling in prostate tumorigenesis to develop new and more effective therapeutic strategies for treating PCa. Recently, a significant niche role of stromal AR in Sonic Hedgehog (Shh) responsive Gli1-lineage cells through stromal-epithelial paracrine interactions has been identified in regulating prostate development and tumorigenesis [17–19]. Here, we summarized the latest discoveries of stromal AR niches and their interactions with prostate epithelia in controlling prostate epithelial differentiation, growth, and regeneration. In combination with emerging clinical and experimental evidence, we also discuss several important and long-term unanswered questions regarding stromal AR niches in promoting prostate epithelial oncogenesis, and tumor development and progression, and highlight future therapeutic strategies for co-targeting both epithelial and stromal AR oncogenic effects for treating advanced PCa.

### Stromal androgen signaling in prostate development, morphogenesis, and growth

The androgen signaling pathway is mediated through the AR and its ligands, testosterone and 5α-dihydrotestosterone [12]. The AR gene is located on the X chromosome; therefore, in non-cancerous conditions (e.g. no amplification), the presence of a single allele in male allows for the direct identification and manifestation of individual mutations [20]. Activation of the AR through the ligand-receptor interaction is essential in early prostate development, morphogenesis, growth, and regeneration [21]. The AR is a member of the steroid hormone receptor superfamily and is a ligand-regulated transcriptional factor [22, 23]. It forms a complex with heat-shock proteins (HSPs) in the cytoplasm when unbound [24, 25]. Upon binding to androgens, the AR dissociates from the HSPs and translocates into the nucleus, where it binds to the androgen response elements on the promoters of target genes to induce their transcription [21]. Emerging evidence has shown that the determination and initiation of prostatic development in both humans and rodents are exclusively dependent upon AR-mediated signaling pathways [1, 26]. During embryogenesis, the AR is initially expressed in the UGM prior to the initiation of prostate budding and morphogenesis, after which its expression extends to the UGE [1, 2, 27]. Mutation of the *Ar* gene completely abolishes the prostate development resulting in testicular feminization (Tfm) in male mice [4]. Intriguingly, early tissue recombination experiments showed that mesenchymal, rather than epithelial, AR action plays a decisive role in controlling UGE development and prostatic gland formation [3, 5]. Further studies using the similar experimental approaches showed that mesenchymal androgen signaling can direct undifferentiated epithelial cells isolated from the bladder or female urogenital sinus to differentiate into functional prostatic epithelium [4, 5, 28]. These findings provide the first line of experimental evidence to demonstrate an essential role of the UGM as a stromal niche in supporting prostatic stem/progenitor-mediated early prostate development, differentiation, and expansion.

In the postnatal prostate, androgen signaling continues to play a crucial role in controlling prostate maturation, growth, and regeneration through mesenchymal-epithelial interactions [6]. The morphogenesis of the mouse prostate complex is completed between postnatal days 15 and 30 (P15-30), and continuing growth and maturation occur during puberty (P25-40) when circulating androgen levels rise [29–31]. Beyond puberty, androgen signaling remains essential in prostate cell differentiation and expansion, as evidenced by prostatic regeneration through repeated cycles of androgen deprivation and replacement [1, 28, 32]. This phenomenon was also observed in experimental rodent models as well as in seasonally breeding animals, affirming the role of androgens in maintaining prostate homeostasis and regenerative capacity [8, 33]. The regulatory role of mesenchymal AR action to support prostatic epithelial growth and homeostasis has been studied using various genetically engineered mouse models (GEMMs) and other relevant approaches. Specifically, as the UGM ultimately differentiates to form prostate stroma, significant research efforts have been focused on understanding the effect of stromal AR expression in prostate epithelial development, maturation, and growth. Selective deletion of AR in mouse stromal fibroblasts (FBs) using the *fibroblast specific protein 1* (*Fsp1*) promoter-driven *Cre* recombinase resulted in a grossly normal prostate in comparison to wild-type controls [34]. In a different GEMM, selective deletion of AR in stromal smooth muscle cells (SMCs) using the *transgelin (Tagln) promoter*-driven Cre recombinase, showed slight pathological changes with loss of infolding structures and a decrease in epithelial proliferation in adult prostate glands [35]. The compound mouse model bearing AR deletion in both stromal FBs and SMCs led to reduced size of the anterior prostate lobes with pathological changes such as impaired branching and partial loss of the infolding glandular structure [36]. It is important to note that the data from the above GEMMs differ from the results of the early tissue recombination assays showing a dominant role of stromal AR expression in prostate embryonic development and morphogenesis [3–5], questioning the role of stromal AR and the cellular properties of prostatic stromal cells that can function as stem cell niches to support prostate epithelial differentiation and growth.

### Aberrant androgen signaling activation promotes prostate tumorigenesis

The activation of the AR through the ligand-receptor interaction forms a central axis for prostate tumorigenesis [7, 8, 10, 11]. Primary PCa cells express the AR and require androgens for their growth and survival [8, 10]. Thus, ADT, directly targeting AR-expressing prostate tumor cells, has been the main, and also initially effective treatment for advanced PCa [16]. However, it eventually fails in nearly all patients, and consequently, patients develop CRPC within 2-3 years after initiating the therapy. Emerging evidence has shown that dysregulated AR signaling directly contributes to CRPC development. Global gene expression profiling shows that *AR* is the only gene that is consistently up-regulated in CRPC samples [37]. Amplification of the *AR* gene is present in one-third of PCa after ADT [38, 39]. Mutations within the *AR* gene and the dysregulation of AR co-regulators have also been identified in a significant portion of CRPC [40, 41]. Additionally, many diverse AR splice variants lacking the ligand-binding domains have been identified in CRPC samples [42, 43], implicating the constitutive activities of these variants in supporting ligand-independent PCa cell growth and progression. These lines of scientific evidence affirm the promotional role of aberrant AR activation in PCa growth, progression, and CRPC development during the course of ADT [11, 44].

To inhibit the re-activation of AR-induced PCa growth during ADT, significant efforts have been devoted to developing more potent AR antagonists and androgen synthesis inhibitors [45]. Whereas these second-generation antagonists and inhibitors showed some clinical effectiveness, they could also induce more diverse CRPC phenotypes and promote tumor progression, worsening clinical outcomes [46]. For example, a subpopulation of AR- and neuroendocrine (NE)-null PCa cells, termed double-null PCa (DNPC), has been observed frequently in patients treated with abiraterone, an androgen synthesis inhibitor and enzalutamide, an AR antagonist, directly contributing to the increased incidence and mortality of metastatic CRPC [47]. To address these new clinical challenges, more efforts are needed to investigate the roles of androgen/AR signaling in both epithelial tumor cells and their niches during current ADT treatments to gain more and deeper insights into the development of new and effective therapeutic strategies for advanced PCa.

### Reciprocal epithelial-stromal interactions regulate prostate tumorigenesis

Emerging evidence has implicated a crucial but unclear role of stromal-epithelial interactions during the course of PCa development and progression [48]. The tumor stroma is a complex mixture of cells that includes FBs, SMCs, and other stromal cell components, also called cancer-associated stroma or reactive stroma [48–50]. Like in other tumor stroma, prostatic tumor stromal cells produce various chemokines, cytokines, and growth factors that act as messengers to regulate epithelial tumor cell growth and progression through reciprocal tumor-stroma interactions [48, 51, 52]. In contrast, normal or benign stromal cells can inhibit malignant epithelial cell proliferation, and reverse the malignant cell properties of tumor cells [53]. Additionally, emerging evidence suggests a critical role of tumor stroma in promoting drug resistance as observed in tumor microenvironment of clinical samples [51, 52]. Elevated levels of hepatocyte growth factor (HGF), insulin-like growth factor 1 (IGF1), and Wnt ligands have been identified in patients who were resistant to tyrosine kinase inhibitors or chemotherapy [54–56]. It has also been reported that increased neuregulin 1, produced in cancer-associated FBs (CAFs), can promote castration-resistance in a mouse model and human prostate organoid cultures [57]. Recently, it has been shown that ADT can activate HGF and canonical Wnt signaling pathways that further induce Exportin 1 (XPO1/CRM1) and ribosomal biogenesis pathways to promote PCa progression and DNPC development in both human PCa samples and related GEMMs [58]. However, it remains poorly understood how prostate cancer stromal cells function as tumor niches to promote hormone refractoriness and tumor progression through ADT.

### Stromal AR signaling supports prostate tumorigenesis

Emerging evidence suggests that AR-mediated signaling pathways through continuous reciprocal stromal-epithelial interactions are essential for PCa initiation and progression [59–62]. The expression of AR has been identified in both PCa cells and their surrounding stromal cells [63]. Activation of AR in prostate epithelium, particularly in luminal cells, can activate AR downstream target gene expression and induce various signaling pathways to promote epithelial morphogenesis and growth [64]. In turn, AR activation in prostate stromal cells not only directly supports stromal cell growth, but also induces the secretion of various paracrine factors that diffuse from the stromal compartment to the epithelium, supporting epithelial cell growth, survival, and differentiation [65]. However, the exact effect of stromal AR in prostate tumorigenesis remained controversial. Reduced AR expression was observed in stromal cells surrounding prostate intraepithelial neoplasia (PIN) and PCa lesions [66–69]. Co-culture of AR-deficient prostatic stromal cells with PCa epithelial cells also appeared to enhance tumor cell growth in both in vitro and xenograft models [67]. However, the compound mice with *AR* deletion in both stromal FBs and SMCs in combination with *Pten* deletion in prostatic epithelia showed a significant delay in PIN development [70]. The exact reasons for the discrepancy of the above experimental results are still unclear. Therefore, more investigations with new and biologically relevant in vivo models and systems should be warranted to determine the biological role of stromal AR as prostate tumor niches during the course of PCa development, progression, and hormone refractoriness.

### Sonic hedgehog signaling pathways in prostate development, growth, and tumorigenesis

Sonic hedgehog (Shh) is one of three mammalian hedgehog proteins and plays a critical role in vertebrate embryonic development and tumorigenesis [71, 72]. The important role of Shh signaling in adult stem cells has been explored in many self-renewing organs, including the prostate [73–75]. Multiple lines of experimental evidence and clinical observations have shown that Shh signaling directly regulates prostatic development, homeostasis, and tumorigenesis through reciprocal epithelial-mesenchymal interactions [76–78]. The Shh growth factor and its downstream effectors, Gli proteins, are expressed in murine prostatic epithelial and mesenchymal cells, respectively, during embryogenesis as well as throughout the adulthood [75, 79]. The expression pattern of Shh and Gli1 in prostate tissues closely correlates the regulatory role of Shh signaling through the paracrine interaction between prostatic epithelial and stromal cells [80–82]. Activation of Shh signaling directly regulates the growth and fate of prostate epithelial cells during the course of prostate early morphogenesis and formation [78, 83]. Additionally, it has been shown that prostatic Gli1-expressing cells possess stromal progenitor properties and enable the repopulation of prostatic stromal cells during androgen depletion and supplementation cycles [75]. However, loss of Gli1 and other Gli protein expression has been shown to be dispensable for mouse prostate development and morphogenesis [75, 79], raising the questions regarding their biological significance in regulating prostate cell differentiation and growth.

Similarly, the expression of Shh and Gli1 was also observed in epithelial and stromal cells in human prostate tumor tissues [84], confirming the paracrine regulations between tumor epithelia and stroma. Overexpression of Shh in PCa cells can elevate stromal Gli1 expression and accelerate tumor growth in the xenograft tumor model [84]. Increased Shh expression was observed in PCa cells cultured in androgen-depleted medium [85, 86]. Combination treatment with inhibitors for the AR and Shh pathways appeared to suppress the growth of CRPC cells more effectively than did treatment with either agent alone in PCa xenograft models [54]. However, co-inhibition of Shh and AR signaling in clinic did not show a clear benefit for CRPC patients [76, 87, 88]. The underlying mechanism for this clinical failure remains unclear, raising a question regarding the tumor niche roles of stromal AR and Shh signaling pathways in regulating prostate epithelial oncogenesis, PCa progression, and CRPC development.

### AR expression in stromal Gli1-expressing cells plays an indispensable role in prostate development, morphogenesis, and regeneration

Recently, a biological role of the AR in prostatic Shh-responsive Gli1-expressing cells was assessed in prostate development, growth, and regeneration using the new and relevant GEMM [18], which bears selective deletion of AR expression in Gli1-expressing cells. The mice displayed diminished prostatic budding and prostate glandular development and formation [18]. Tissue recombination assays using UGM containing AR-deficient Gli1-expressing cells from the above mice combined with wild-type UGE failed to develop normal prostate glandular tissue in the presence of androgens [18]. These data implicate an indispensable role of AR expression in mesenchymal Gli1-expressing cells in prostate early development. Additionally, prepubescent deletion of AR expression in Gli1-expressing cells on postnatal day 14 (P14) in the above mouse model also showed a severe impairment of prostate prepubescent morphogenesis and differentiation, and pubertal growth [18]. Moreover, the deletion of AR expression in Gli1-expressing cells in the adult prostates (P56) significantly impaired prostatic epithelial regeneration through androgen depletion and supplementation cycles. Single-cell RNA sequencing (scRNA-seq) analyses of Gli1-activated mGFP expression stromal cells, which include *Gli1*-expressing cells and their descendants, further referred to as *Gli1*-lineage cells, showed that AR loss disrupts androgen signaling-initiated stromal-epithelial paracrine interactions, and elevates Shh and other developmental and metabolic stress signaling pathways to inhibit prostatic epithelial growth [19]. Further analyses of adjacent prostatic epithelial cells in the above AR-deficient samples revealed a reduction in luminal epithelial clusters. Single-cell trajectory analyses further identified an aberrant differentiation fate of prostatic epithelial cells in AR-deficient mouse samples. The in vivo recombination assays with purified AR-deficient stromal *Gli1*-lineage cells isolated from the above adult mice in combination with wild-type adult prostatic epithelial cells failed to develop normal prostatic epithelia. These lines of experimental evidence differ significantly from previous studies with GEMMs bearing AR deletion in prostate FBs and SMCs [34]. It implicates a novel and indispensable role of stromal AR in Gli1-lineage cells as stromal niches that support prostate epithelial development, differentiation, pubertal growth, and regeneration [18, 19].

### Stromal androgen signaling in Gli1-lineage cells functions as tumor niches to support prostatic basal epithelial progenitor-initiated oncogenesis

**Fig. 1:**
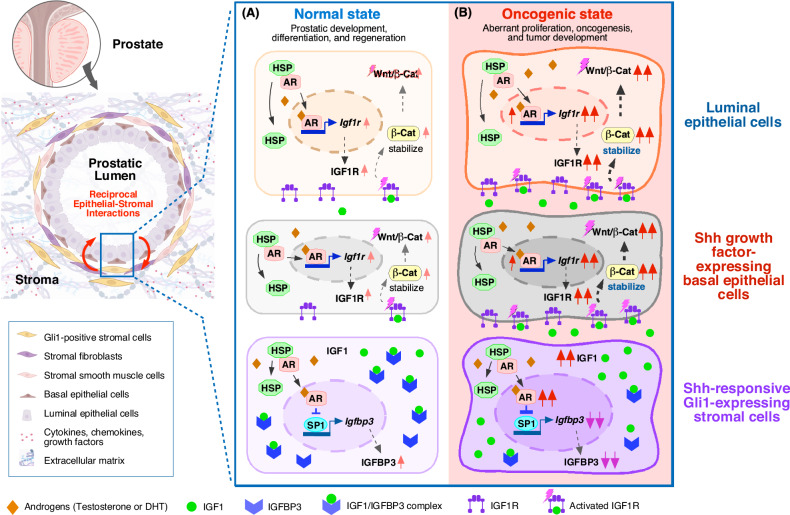
Androgen signaling in stromal Gli1-lineage cells plays a niche role in normal and oncogenic prostate tissues. **A** In normal prostatic state, AR activation of Shh-responsive, Gli1-lineage stromal cells through the binding of androgens stimulates IGF1R expression in prostatic basal epithelial cells and attenuates SP1-regulated IGFBP3 expression in stromal cells, which in return increases IGF1 bioavailability. Subsequent activation of IGF1 and Wnt/β-catenin axes in basal and luminal epithelial cells promotes prostate development, differentiation, and regeneration. **B** In prostatic oncogenic state, non-physiologic, aberrant activation of AR and/or IGF1 axes can lead to dysregulated activation of IGF1 and Wnt/β-catenin axes in basal and luminal epithelial cells, promoting prostatic epithelial oncogenesis.

Over the past decades, significant effort has been devoted to studying prostate tumor cell-intrinsic mechanisms for PCa initiation, progression, and CRPC development [13–15]. However, emerging evidence also implicates a non-autonomous role of androgen/AR signaling in prostatic stroma to support PCa tumorigenesis [47, 57, 89, 90]. As such, the niche role of stromal AR in Gli1-lineage cells to promote prostate tumor initiation and progression has been assessed using newly generated mouse models and human PCa datasets and samples [17] (see Fig. 1). In the compound mouse model, selective deletion of stromal AR expression in Gli1-lineage cells impaired transgenic Myc-induced prostatic epithelial oncogenesis and tumor development [17]. Specifically, significant reduction of Myc-positive basal atypical cells was observed in the above mouse models. Given the proximity of prostate basal epithelial cells to stromal cells, these findings suggest that stromal AR expression in the Gli1-lineage cell niche facilitates the oncogenic initiation driven by prostatic basal progenitors. ScRNA-seq analyses of *hMycTg-*positive basal atypical cells isolated from the above compound mice identified a remarkable reduction in both IGF1R and Wnt/β-catenin signaling activation. Because aberrant IGF1 and Wnt activation has been shown to directly induce prostate oncogenesis and PCa development [91], these findings implicate stromal AR in Gli1-lineage cells acting as tumor niches to support prostate epithelial oncogenesis through IGF1/Wnt activation. Intriguingly, scRNA-seq analyses showed that *Igfbp3*, insulin-like growth factor-binding protein 3 (IGFBP3), was one of the most highly expressed genes in AR-deficient Gli1-lineage stromal cells. An inverse correlation between *Ar* and *Igfbp3* expression was further confirmed in Gli1-lineage cells using scRNA-seq analyses and other experimental approaches [17]. It has been shown that *Igfbp3* transcription is mainly regulated through Sp1 transcription factor [92], and that the AR can interfere with Sp1 binding to its target promoters and represses Sp1-mediated transcription [93, 94]. Accordingly, ChIP-qPCR analyses identified increased occupancy of Sp1 within the *Igfbp3* promoter region in sorted AR-deficient Gli1-lineage cells in comparison to Gli1-lineage cells from control mice [95], affirming the repressive role of AR on the recruitment of Sp1 to the *Igfbp3* promoter. As the primary role of IGFBP3 is to bind and transport IGF1 through which can limit bioavailable IGF1, these data uncover a new regulatory role of stromal AR in regulating IGF1 activity through controlling IGFBP3 expression in prostatic stromal Gli1-lineage cells. As summarized in Fig. 1, these experimental data demonstrate a regulatory mechanism underlying androgen/AR signaling pathways to support prostatic epithelial oncogenesis as well as PCa development and growth through the activation of IGF1 axes in stromal cells.

## Remaining questions and future prospects

While a significant role for stromal androgen/AR signaling in prostate development, differentiation, and regeneration has been suggested in the past, the cellular properties and characteristics of prostatic stromal niches and the regulatory mechanisms governing the reciprocal interaction between stromal and epithelial cells were poorly understood until recently. As summarized in this review, identification of an indispensable role of stromal AR in Gli1-lineage cells in supporting prostate embryonic epithelium development, prepubertal morphogenesis, and pubertal growth, as well as prostatic epithelial oncogenesis and tumor development provides new insight into our current understanding on stromal niche roles of androgen signaling. Meanwhile, these lines of newly emerged scientific evidence also raise several important but currently unknown questions in the field. First, since previous studies have shown the expression of Gli1 and other Gli transcription factors are dispensable for prostate development and morphogenesis, identifying an indispensable role of AR expression in Gli1-lineage cells implicates a new and unique regulatory mechanism underlying AR and Gli1 interactions for prostate development, maturation, and tumorigenesis. As such, the implications of the AR and Gli1 interactions through stromal and epithelial cells in both normal and tumor status should be further investigated. Second, given that previous studies have shown that selective deletion of AR either in prostatic FBs or SMCs alone or both only resulted in modest pubertal prostate growth defects [34, 36], it is possible that the population of prostatic stromal Gli1-expressing cells is different from the general prostate FBs and SMCs. Therefore, identification of the cellular properties of the prostatic Gli1-expressing cells will be extremely important to fully understand the precise identity and role of prostatic stem niche cells. Third, a significant increase in IGFBP3 expression in AR-deficient Gli1-lineage cells suggests a regulatory role for AR in Gli1-lineage niche cells to support PCa growth through regulating IGF1 axes. Thus far, it is unclear whether this regulation is divergent during ADT and related tumor progression, which may lead to aberrant activation of IGF1 to promote tumor progression and hormone refractoriness, resulting in CRPC development. Moreover, multiple lines of clinical and experimental evidence have demonstrated the biological roles of androgen signaling in both myeloid and lymphoid cells within the prostate stroma [96–98]. The effects of androgen signaling on these cells, along with alterations in the cytokine environment, can reshape the tumor microenvironment, facilitating tumor progression and resistance to immunotherapies [99]. As such, further investigations are warranted to explore in the interactions between androgen signaling and immune cells. Finally, building on the critical role of stromal AR signaling in PCa, future therapeutic strategies could focus on co-targeting both epithelial and stromal androgen and AR mediated oncogenic pathways. Stromal AR signaling, particularly within Gli1-lineage cells, may significantly contribute to therapy resistance by supporting epithelial cell survival and tumor growth during ADT. Therefore, targeting both tumor epithelial AR and stromal AR signaling could simultaneously disrupt the tumor microenvironment and inhibit epithelial tumor growth. Such an approach may offer a more comprehensive strategy for treating advanced prostate cancer. Continued research in this area is essential to gain deeper insights into these mechanisms and develop more effective therapies by co-targeting these aberrant AR signaling pathways.

## References

[1] Cunha GR, Donjacour AA, Cooke PS, Mee S, Bigsby RM, Higgins SJ, et al. The endocrinology and developmental biology of the prostate. Endocr Rev. 1987;8:338–62. 3308446 10.1210/edrv-8-3-338

[2] Cooke PS, Young P, Cunha GR. Androgen receptor expression in developing male reproductive organs. Endocrinology. 1991;128:2867–73. 2036966 10.1210/endo-128-6-2867

[3] Cunha GR, Lung B. The possible influence of temporal factors in androgenic responsiveness of urogenital tissue recombinants from wild-type and androgen-insensitive (Tfm) mice. J Exp Zool. 1978;205:181–93. 681909 10.1002/jez.1402050203

[4] Cunha GR, Chung LW. Stromal-epithelial interactions–I. Induction of prostatic phenotype in urothelium of testicular feminized (Tfm/y) mice. J Steroid Biochem. 1981;14:1317–24. 6460136 10.1016/0022-4731(81)90338-1

[5] Cunha GR. Androgenic effects upon prostatic epithelium are mediated via trophic influences from stroma. Prog Clin Biol Res. 1984;145:81–102. 6371832

[6] Brennen WN, Isaacs JT. Mesenchymal stem cells and the embryonic reawakening theory of BPH. Nat Rev Urol. 2018;15:703–15. 30214054 10.1038/s41585-018-0087-9 PMC7050678

[7] Gelmann EP. Molecular biology of the androgen receptor. J Clin Oncol. 2002;20:3001–15. 12089231 10.1200/JCO.2002.10.018

[8] Kyprianou N, Isaacs JT. Activation of programmed cell death in the rat ventral prostate after castration. Endocrinology. 1988;122:552–62. 2828003 10.1210/endo-122-2-552

[9] Rawla P. Epidemiology of Prostate Cancer. World J Oncol. 2019;10:63–89. 31068988 10.14740/wjon1191 PMC6497009

[10] Abate-Shen C, Shen MM. Molecular genetics of prostate cancer. Genes Dev. 2000;14:2410–34. 11018010 10.1101/gad.819500

[11] Culig Z, Klocker H, Bartsch G, Steiner H, Hobisch A. Androgen receptors in prostate cancer. J Urol. 2003;170:1363–9. 14501770 10.1097/01.ju.0000075099.20662.7f

[12] Zhou ZX, Wong CI, Sar M, Wilson EM. The androgen receptor: an overview. Recent Prog Horm Res. 1994;49:249–74. 8146426 10.1016/b978-0-12-571149-4.50017-9

[13] Abida W, Cyrta J, Heller G, Prandi D, Armenia J, Coleman I, et al. Genomic correlates of clinical outcome in advanced prostate cancer. Proc Natl Acad Sci USA. 2019;116:11428–36. 31061129 10.1073/pnas.1902651116 PMC6561293

[14] Robinson D, Van Allen EM, Wu Y-M, Schultz N, Lonigro RJ, Mosquera J-M, et al. Integrative clinical genomics of advanced prostate cancer. Cell. 2015;161:1215–28. 26000489 10.1016/j.cell.2015.05.001 PMC4484602

[15] Wu HC, Hsieh JT, Gleave ME, Brown NM, Pathak S, Chung LW. Derivation of androgen-independent human LNCaP prostatic cancer cell sublines: role of bone stromal cells. Int J Cancer. 1994;57:406–12. 8169003 10.1002/ijc.2910570319

[16] Huggins C, Hodges CV. Studies on prostatic cancer: I. The effect of castration, of estrogen and of androgen injection on serum phosphatases in metastatic carcinoma of the prostate. 1941. J Urol. 2002;168:9–12. 12050481 10.1016/s0022-5347(05)64820-3

[17] Hiroto A, Kim WK, Pineda A, He Y, Lee DH, Le V, et al. Stromal androgen signaling acts as tumor niches to drive prostatic basal epithelial progenitor-initiated oncogenesis. Nat Commun. 2022;13:6552. 36323713 10.1038/s41467-022-34282-w PMC9630272

[18] Le V, He Y, Aldahl J, Hooker E, Yu EJ, Olson A, et al. Loss of androgen signaling in mesenchymal sonic hedgehog responsive cells diminishes prostate development, growth, and regeneration. PLoS Genet. 2020;16:e1008588. 31929563 10.1371/journal.pgen.1008588 PMC6980684

[19] Olson AW, Le V, Wang J, Hiroto A, Kim WK, Lee D-H, et al. Stromal androgen and hedgehog signaling regulates stem cell niches in pubertal prostate development. Development. 2021;148:dev199738. 10.1242/dev.199738 PMC8497774 34427305

[20] Shafi AA, Yen AE, Weigel NL. Androgen receptors in hormone-dependent and castration-resistant prostate cancer. Pharm Ther. 2013;140:223–38. 10.1016/j.pharmthera.2013.07.003 23859952

[21] Jenster G, van der Korput HA, van Vroonhoven C, van der Kwast TH, Trapman J, Brinkmann AO. Domains of the human androgen receptor involved in steroid binding, transcriptional activation, and subcellular localization. Mol Endocrinol. 1991;5:1396–404. 1775129 10.1210/mend-5-10-1396

[22] Chang CS, Kokontis J, Liao ST. Molecular cloning of human and rat complementary DNA encoding androgen receptors. Science. 1988;240:324–6. 3353726 10.1126/science.3353726

[23] Lubahn DB, Joseph DR, Sar M, Tan J, Higgs HN, Larson RE, et al. The human androgen receptor: complementary deoxyribonucleic acid cloning, sequence analysis and gene expression in prostate. Mol Endocrinol. 1988;2:1265–75. 3216866 10.1210/mend-2-12-1265

[24] Sanchez ER, Faber LE, Henzel WJ, Pratt WB. The 56-59-kilodalton protein identified in untransformed steroid receptor complexes is a unique protein that exists in cytosol in a complex with both the 70- and 90-kilodalton heat shock proteins. Biochemistry. 1990;29:5145–52. 2378870 10.1021/bi00473a021

[25] Sullivan WP, Vroman BT, Bauer VJ. Isolation of steroid receptor binding protein from chicken oviduct and production of monoclonal antibodies. J Steroid Biochem Mol Biol. 1992;43:37–41. 4052391 10.1021/bi00336a060

[26] Prins GS, Putz O. Molecular signaling pathways that regulate prostate gland development. Differentiation. 2008;76:641–59. 18462433 10.1111/j.1432-0436.2008.00277.x PMC2824174

[27] Takeda H, Chang C. Immunohistochemical and in-situ hybridization analysis of androgen receptor expression during the development of the mouse prostate gland. J Endocrinol. 1991;129:83–89. 2030333 10.1677/joe.0.1290083

[28] Chung LW, Cunha GR. Stromal-epithelial interactions: II. Regulation of prostatic growth by embryonic urogenital sinus mesenchyme. Prostate. 1983;4:503–11. 6889194 10.1002/pros.2990040509

[29] Hayashi N, Sugimura Y, Kawamura J, Donjacour AA, Cunha GR. Morphological and functional heterogeneity in the rat prostatic gland. Biol Reprod. 1991;45:308–21. 1786296 10.1095/biolreprod45.2.308

[30] Staack A, Donjacour AA, Brody J, Cunha GR, Carroll P. Mouse urogenital development: a practical approach. Differentiation. 2003;71:402–13. 12969333 10.1046/j.1432-0436.2003.7107004.x

[31] Sugimura Y, Cunha GR, Donjacour AA. Morphogenesis of ductal networks in the mouse prostate. Biol Reprod. 1986;34:961–71. 3730488 10.1095/biolreprod34.5.961

[32] Cunha GR, Donjacour AA, Sugimara Y. Stromal-epithelial interactions and heterogeneity of proliferative activity within the prostate. Biochem Cell Biol. 1986;64:608–14. 3741678 10.1139/o86-084

[33] Salama G, Noirot O, Bataille V, Malavaud S, Rebillard X, Villers A, et al. Seasonality of serum prostate-specific antigen levels: a population-based study. Eur Urol. 2007;52:708–14. 17174467 10.1016/j.eururo.2006.11.042

[34] Yu S, Yeh CR, Niu Y, Chang HC, Tsai YC, Moses HL, et al. Altered prostate epithelial development in mice lacking the androgen receptor in stromal fibroblasts. Prostate. 2012;72:437–49. 21739465 10.1002/pros.21445 PMC4402036

[35] Yu S, Zhang C, Lin CC, Niu Y, Lai KP, Chang HC, et al. Altered prostate epithelial development and IGF-1 signal in mice lacking the androgen receptor in stromal smooth muscle cells. Prostate. 2011;71:517–24. 20945497 10.1002/pros.21264 PMC3037429

[36] Lai KP, Yamashita S, Vitkus S, Shyr CR, Yeh S, Chang C. Suppressed prostate epithelial development with impaired branching morphogenesis in mice lacking stromal fibromuscular androgen receptor. Mol Endocrinol. 2012;26:52–66. 22135068 10.1210/me.2011-1189 PMC3248327

[37] Chen CD, Welsbie DS, Tran C, Baek SH, Chen R, Vessella R, et al. Molecular determinants of resistance to antiandrogen therapy. Nat Med. 2004;10:33–39. 14702632 10.1038/nm972

[38] Koivisto P, Kononen J, Palmberg C, Tammela T, Hyytinen E, Isola J, et al. Androgen receptor gene amplification: a possible molecular mechanism for androgen deprivation therapy failure in prostate cancer. Cancer Res. 1997;57:314–9. 9000575

[39] Ruizeveld de Winter JA, Janssen PJ, Sleddens HM, Verleun-Mooijman MC, Trapman J, Brinkmann AO, et al. Androgen receptor status in localized and locally progressive hormone refractory human prostate cancer. Am J Pathol. 1994;144:735–46. 7512791 PMC1887232

[40] Taplin ME, Bubley GJ, Shuster TD, Frantz ME, Spooner AE, Ogata GK, et al. Mutation of the androgen-receptor gene in metastatic androgen- independent prostate cancer. N Engl J Med. 1995;332:1393–8. 7723794 10.1056/NEJM199505253322101

[41] Gaddipati JP, McLeod DG, Heidenberg HB, Sesterhenn IA, Finger MJ, Moul JW, et al. Frequent detection of codon 877 mutation in the androgen receptor gene in advanced prostate cancers. Cancer Res. 1994;54:2861–4. 8187068

[42] Guo Z, Yang X, Sun F, Jiang R, Linn DE, Chen H, et al. A novel androgen receptor splice variant is up-regulated during prostate cancer progression and promotes androgen depletion-resistant growth. Cancer Res. 2009;69:2305–13. 19244107 10.1158/0008-5472.CAN-08-3795 PMC2672822

[43] Paschalis A, Sharp A, Welti JC, Neeb A, Raj GV, Luo J, et al. Alternative splicing in prostate cancer. Nat Rev Clin Oncol. 2018;15:663–75. 30135575 10.1038/s41571-018-0085-0

[44] Culig Z, Hobisch A, Bartsch G, Klocker H. Androgen receptor–an update of mechanisms of action in prostate cancer. Urol Res. 2000;28:211–9. 11011957 10.1007/s002400000111

[45] Titus MA, Gregory CW, Ford OH 3rd, Schell MJ, Maygarden SJ, Mohler JL, et al. Steroid 5alpha-reductase isozymes I and II in recurrent prostate cancer. Clin Cancer Res. 2005;11:4365–71. 15958619 10.1158/1078-0432.CCR-04-0738

[46] Li Q, Deng Q, Chao HP, Liu X, Lu Y, Lin K, et al. Linking prostate cancer cell AR heterogeneity to distinct castration and enzalutamide responses. Nat Commun. 2018;9:3600. 30190514 10.1038/s41467-018-06067-7 PMC6127155

[47] Bluemn EG, Coleman IM, Lucas JM, Coleman RT, Hernandez-Lopez S, Tharakan R, et al. Androgen Receptor Pathway-Independent Prostate Cancer Is Sustained through FGF Signaling. Cancer Cell. 2017;32:474–489.e476. 29017058 10.1016/j.ccell.2017.09.003 PMC5750052

[48] Sasaki T, Franco OE, Hayward SW. Interaction of prostate carcinoma-associated fibroblasts with human epithelial cell lines in vivo. Differentiation. 2017;96:40–48. 28779656 10.1016/j.diff.2017.07.002 PMC5669818

[49] Rowley DR. What might a stromal response mean to prostate cancer progression? Cancer Metastasis Rev. 1998;17:411–9. 10453285 10.1023/a:1006129420005

[50] Tuxhorn JA, Ayala GE, Smith MJ, Smith VC, Dang TD, Rowley DR. Reactive stroma in human prostate cancer: induction of myofibroblast phenotype and extracellular matrix remodeling. Clin Cancer Res. 2002;8:2912–23. 12231536

[51] Zhang XH, Jin X, Malladi S, Zou Y, Wen YH, Brogi E, et al. Selection of bone metastasis seeds by mesenchymal signals in the primary tumor stroma. Cell. 2013;154:1060–73. 23993096 10.1016/j.cell.2013.07.036 PMC3974915

[52] Klemm F, Joyce JA. Microenvironmental regulation of therapeutic response in cancer. Trends Cell Biol. 2015;25:198–213. 25540894 10.1016/j.tcb.2014.11.006 PMC5424264

[53] Hayashi N, Cunha GR. Mesenchyme-induced changes in the neoplastic characteristics of the Dunning prostatic adenocarcinoma. Cancer Res. 1991;51:4924–30. 1893381

[54] Sun Y, Campisi J, Higano C, Beer TM, Porter P, Coleman I, et al. Treatment-induced damage to the tumor microenvironment promotes prostate cancer therapy resistance through WNT16B. Nat Med. 2012;18:1359–68. 22863786 10.1038/nm.2890 PMC3677971

[55] Gilbert LA, Hemann MT. DNA damage-mediated induction of a chemoresistant niche. Cell. 2010;143:355–66. 21029859 10.1016/j.cell.2010.09.043 PMC2972353

[56] Crawford Y, Kasman I, Yu L, Zhong C, Wu X, Modrusan Z, et al. PDGF-C mediates the angiogenic and tumorigenic properties of fibroblasts associated with tumors refractory to anti-VEGF treatment. Cancer Cell. 2009;15:21–34. 19111878 10.1016/j.ccr.2008.12.004

[57] Zhang Z, Karthaus WR, Lee YS, Gao VR, Wu C, Russo JW, et al. Tumor Microenvironment-Derived NRG1 Promotes Antiandrogen Resistance in Prostate Cancer. Cancer Cell. 2020;38:279–296.e279. 32679108 10.1016/j.ccell.2020.06.005 PMC7472556

[58] Kim WK, Buckley AJ, Lee DH, Hiroto A, Nenninger CH, Olson AW, et al. Androgen deprivation induces double-null prostate cancer via aberrant nuclear export and ribosomal biogenesis through HGF and Wnt activation. Nat Commun. 2024;15:1231. 38336745 10.1038/s41467-024-45489-4 PMC10858246

[59] Nieto CM, Rider LC, Cramer SD. Influence of stromal-epithelial interactions on androgen action. Endocr Relat cancer. 2014;21:T147–160. 24872510 10.1530/ERC-14-0138

[60] Ricke EA, Williams K, Lee YF, Couto S, Wang Y, Hayward SW, et al. Androgen hormone action in prostatic carcinogenesis: stromal androgen receptors mediate prostate cancer progression, malignant transformation and metastasis. Carcinogenesis. 2012;33:1391–8. 22535887 10.1093/carcin/bgs153 PMC3499049

[61] Wang H, Li N, Liu Q, Guo J, Pan Q, Cheng B, et al. Antiandrogen treatment induces stromal cell reprogramming to promote castration resistance in prostate cancer. Cancer Cell. 2023;41:1345–1362.e1349. 37352863 10.1016/j.ccell.2023.05.016

[62] Yu S, Xia S, Yang D, Wang K, Yeh S, Gao Z, et al. Androgen receptor in human prostate cancer-associated fibroblasts promotes prostate cancer epithelial cell growth and invasion. Med Oncol. 2013;30:674. 23925662 10.1007/s12032-013-0674-9

[63] Ricciardelli C, Choong CS, Buchanan G, Vivekanandan S, Neufing P, Stahl J, et al. Androgen receptor levels in prostate cancer epithelial and peritumoral stromal cells identify non-organ confined disease. Prostate. 2005;63:19–28. 15378523 10.1002/pros.20154

[64] Hayward SW, Haughney PC, Rosen MA, Greulich KM, Weier HU, Dahiya R, et al. Interactions between adult human prostatic epithelium and rat urogenital sinus mesenchyme in a tissue recombination model. Differ Res Biol Diversity. 1998;63:131–40. 10.1046/j.1432-0436.1998.6330131.x 9697307

[65] Kurita T, Wang YZ, Donjacour AA, Zhao C, Lydon JP, O’Malley BW, et al. Paracrine regulation of apoptosis by steroid hormones in the male and female reproductive system. Cell Death Differ. 2001;8:192–200. 11313721 10.1038/sj.cdd.4400797

[66] Henshall SM, Quinn DI, Lee CS, Head DR, Golovsky D, Brenner PC, et al. Altered expression of androgen receptor in the malignant epithelium and adjacent stroma is associated with early relapse in prostate cancer. Cancer Res. 2001;61:423–7. 11212224

[67] Li Y, Li CX, Ye H, Chen F, Melamed J, Peng Y, et al. Decrease in stromal androgen receptor associates with androgen- independent disease and promotes prostate cancer cell proliferation and invasion. J Cell Mol Med. 2008;12:2790–8. 18266956 10.1111/j.1582-4934.2008.00279.x PMC3828892

[68] Olapade-Olaopa EO, MacKay EH, Taub NA, Sandhu DP, Terry TR, Habib FK. Malignant transformation of human prostatic epithelium is associated with the loss of androgen receptor immunoreactivity in the surrounding stroma. Clin Cancer Res. 1999;5:569–76. 10100708

[69] Wikström P, Marusic J, Stattin P, Bergh A. Low stroma androgen receptor level in normal and tumor prostate tissue is related to poor outcome in prostate cancer patients. Prostate. 2009;69:799–809. 19189305 10.1002/pros.20927

[70] Lai KP, Yamashita S, Huang CK, Yeh S, Chang C. Loss of stromal androgen receptor leads to suppressed prostate tumourigenesis via modulation of pro-inflammatory cytokines/chemokines. EMBO Mol Med. 2012;4:791–807. 22745041 10.1002/emmm.201101140 PMC3494077

[71] Briscoe J, Thérond PP. The mechanisms of Hedgehog signalling and its roles in development and disease. Nat Rev Mol Cell Biol. 2013;14:416–29. 23719536 10.1038/nrm3598

[72] Jiang J, Hui CC. Hedgehog signaling in development and cancer. Dev Cell. 2008;15:801–12. 19081070 10.1016/j.devcel.2008.11.010 PMC6443374

[73] Ahn S, Joyner AL. In vivo analysis of quiescent adult neural stem cells responding to Sonic hedgehog. Nature. 2005;437:894–7. 16208373 10.1038/nature03994

[74] Kugler MC, Joyner AL, Loomis CA, Munger JS. Sonic hedgehog signaling in the lung. From development to disease. Am J Respir Cell Mol Biol. 2015;52:1–13. 25068457 10.1165/rcmb.2014-0132TR PMC4370254

[75] Peng YC, Levine CM, Zahid S, Wilson EL, Joyner AL. Sonic hedgehog signals to multiple prostate stromal stem cells that replenish distinct stromal subtypes during regeneration. Proc Natl Acad Sci USA. 2013;110:20611–6. 24218555 10.1073/pnas.1315729110 PMC3870668

[76] Peng YC, Joyner AL. Hedgehog signaling in prostate epithelial-mesenchymal growth regulation. Dev Biol. 2015;400:94–104. 25641695 10.1016/j.ydbio.2015.01.019 PMC4361250

[77] Shaw A, Bushman W. Hedgehog signaling in the prostate. J Urol. 2007;177:832–8. 17296352 10.1016/j.juro.2006.10.061

[78] Bushman W. Hedgehog Signaling in Prostate Development, Regeneration and Cancer. J Dev Biol. 2016;4:30. 10.3390/jdb4040030 PMC5831806 29615593

[79] Doles J, Cook C, Shi X, Valosky J, Lipinski R, Bushman W. Functional compensation in Hedgehog signaling during mouse prostate development. Dev Biol. 2006;295:13–25. 16707121 10.1016/j.ydbio.2005.12.002

[80] Lamm ML, Catbagan WS, Laciak RJ, Barnett DH, Hebner CM, Gaffield W, et al. Sonic hedgehog activates mesenchymal Gli1 expression during prostate ductal bud formation. Dev Biol. 2002;249:349–66. 12221011 10.1006/dbio.2002.0774

[81] Scadden DT. The stem-cell niche as an entity of action. Nature. 2006;441:1075–9. 16810242 10.1038/nature04957

[82] Roberts KJ, Kershner AM, Beachy PA. The Stromal Niche for Epithelial Stem Cells: A Template for Regeneration and a Brake on Malignancy. Cancer Cell. 2017;32:404–10. 29017054 10.1016/j.ccell.2017.08.007 PMC5679442

[83] Podlasek CA, Barnett DH, Clemens JQ, Bak PM, Bushman W. Prostate development requires Sonic hedgehog expressed by the urogenital sinus epithelium. Dev Biol. 1999;209:28–39. 10208740 10.1006/dbio.1999.9229

[84] Fan L, Pepicelli CV, Dibble CC, Catbagan W, Zarycki JL, Laciak R, et al. Hedgehog signaling promotes prostate xenograft tumor growth. Endocrinology. 2004;145:3961–70. 15132968 10.1210/en.2004-0079

[85] Azoulay S, Terry S, Chimingqi M, Sirab N, Faucon H, Gil Diez de Medina S, et al. Comparative expression of Hedgehog ligands at different stages of prostate carcinoma progression. J Pathol. 2008;216:460–70. 18825689 10.1002/path.2427

[86] Chen M, Tanner M, Levine AC, Levina E, Ohouo P, Buttyan R. Androgenic regulation of hedgehog signaling pathway components in prostate cancer cells. Cell Cycle. 2009;8:149–57. 19158486 10.4161/cc.8.1.7532 PMC2633936

[87] Cortes JE, Gutzmer R, Kieran MW, Solomon JA. Hedgehog signaling inhibitors in solid and hematological cancers. Cancer Treat Rev. 2019;76:41–50. 31125907 10.1016/j.ctrv.2019.04.005

[88] Maughan BL, Suzman DL, Luber B, Wang H, Glavaris S, Hughes R, et al. Pharmacodynamic study of the oral hedgehog pathway inhibitor, vismodegib, in patients with metastatic castration-resistant prostate cancer. Cancer Chemother Pharmacol. 2016;78:1297–304. 27826729 10.1007/s00280-016-3191-7 PMC5695229

[89] Levesque C, Nelson PS. Cellular Constituents of the Prostate Stroma: Key Contributors to Prostate Cancer Progression and Therapy Resistance. Cold Spring Harbor Perspect Med. 2018;8:a030510. 10.1101/cshperspect.a030510 PMC5681439 28490538

[90] Shiao SL, Chu GC, Chung LW. Regulation of prostate cancer progression by the tumor microenvironment. Cancer Lett. 2016;380:340–8. 26828013 10.1016/j.canlet.2015.12.022 PMC5317350

[91] Kim WK, Olson AW, Mi J, Wang J, Lee DH, Le V, et al. Aberrant androgen action in prostatic progenitor cells induces oncogenesis and tumor development through IGF1 and Wnt axes. Nat Commun. 2022;13:4364. 35902588 10.1038/s41467-022-32119-0 PMC9334353

[92] Choi HS, Lee JH, Park JG, Lee YI. Trichostatin A, a histone deacetylase inhibitor, activates the IGFBP-3 promoter by upregulating Sp1 activity in hepatoma cells: alteration of the Sp1/Sp3/HDAC1 multiprotein complex. Biochem Biophys Res Commun. 2002;296:1005–12. 12200149 10.1016/s0006-291x(02)02001-6

[93] Curtin D, Jenkins S, Farmer N, Anderson AC, Haisenleder DJ, Rissman E, et al. Androgen suppression of GnRH-stimulated rat LHbeta gene transcription occurs through Sp1 sites in the distal GnRH-responsive promoter region. Mol Endocrinol. 2001;15:1906–17. 11682622 10.1210/mend.15.11.0723

[94] Verras M, Lee J, Xue H, Li TH, Wang Y, Sun Z. The androgen receptor negatively regulates the expression of c-Met: implications for a novel mechanism of prostate cancer progression. Cancer Res. 2007;67:967–75. 17283128 10.1158/0008-5472.CAN-06-3552

[95] Wyce A, Bai Y, Nagpal S, Thompson CC. Research Resource: The androgen receptor modulates expression of genes with critical roles in muscle development and function. Mol Endocrinol. 2010;24:1665–74. 20610535 10.1210/me.2010-0138 PMC5417449

[96] Vancolen S, Sebire G, Robaire B. Influence of androgens on the innate immune system. Andrology. 2023;11:1237–44. 36840517 10.1111/andr.13416

[97] Gubbels Bupp MR, Jorgensen TN. Androgen-Induced Immunosuppression. Front Immunol. 2018;9:794. 29755457 10.3389/fimmu.2018.00794 PMC5932344

[98] Guan X, Polesso F, Wang C, Sehrawat A, Hawkins RM, Murray SE, et al. Androgen receptor activity in T cells limits checkpoint blockade efficacy. Nature. 2022;606:791–6. 35322234 10.1038/s41586-022-04522-6 PMC10294141

[99] Hu YM, Zhao F, Graff JN, Chen C, Zhao X, Thomas GV *et al*. Androgen receptor activity inversely correlates with immune cell infiltration and immunotherapy response across multiple cancer lineages. *bioRxiv* 2024.

